# Rates of Gyrase Supercoiling and Transcription Elongation Control Supercoil Density in a Bacterial Chromosome

**DOI:** 10.1371/journal.pgen.1002845

**Published:** 2012-08-16

**Authors:** Nikolay Rovinskiy, Andrews Akwasi Agbleke, Olga Chesnokova, Zhenhua Pang, N. Patrick Higgins

**Affiliations:** 1 Department of Biochemistry and Molecular Genetics, University of Alabama at Birmingham, Birmingham, Alabama, United States of America; 2 Cathay Industrial Biotech, Shanghai, China; Universidad de Sevilla, Spain

## Abstract

Gyrase catalyzes negative supercoiling of DNA in an ATP-dependent reaction that helps condense bacterial chromosomes into a compact interwound “nucleoid.” The supercoil density (σ) of prokaryotic DNA occurs in two forms. Diffusible supercoil density (σ_D_) moves freely around the chromosome in 10 kb domains, and constrained supercoil density (σ_C_) results from binding abundant proteins that bend, loop, or unwind DNA at many sites. Diffusible and constrained supercoils contribute roughly equally to the total *in vivo* negative supercoil density of WT cells, so σ = σ_C_+σ_D_. Unexpectedly, *Escherichia coli* chromosomes have a 15% higher level of σ compared to *Salmonella enterica*. To decipher critical mechanisms that can change diffusible supercoil density of chromosomes, we analyzed strains of *Salmonella* using a 9 kb “supercoil sensor” inserted at ten positions around the genome. The sensor contains a complete Lac operon flanked by directly repeated resolvase binding sites, and the sensor can monitor both supercoil density and transcription elongation rates in WT and mutant strains. RNA transcription caused (−) supercoiling to increase upstream and decrease downstream of highly expressed genes. Excess upstream supercoiling was relaxed by Topo I, and gyrase replenished downstream supercoil losses to maintain an equilibrium state. Strains with TS gyrase mutations growing at permissive temperature exhibited significant supercoil losses varying from 30% of WT levels to a total loss of σ_D_ at most chromosome locations. Supercoil losses were influenced by transcription because addition of rifampicin (Rif) caused supercoil density to rebound throughout the chromosome. Gyrase mutants that caused dramatic supercoil losses also reduced the transcription elongation rates throughout the genome. The observed link between RNA polymerase elongation speed and gyrase turnover suggests that bacteria with fast growth rates may generate higher supercoil densities than slow growing species.

## Author Summary

A 9-kb module called the “supercoil sensor” was used to measure supercoil density at 10 positions in the 4.8-Mb *Salmonella* Typhimurium chromosome. The sensor includes a Lac operon flanked by a pair of directly repeated DNA–binding sites for the γδ recombinase. Measurements of chromosomal supercoil levels and the RNA polymerase elongation rates were made at various positions within the 6 potential macrodomains of the chromosome. Transcription and gyrase catalytic rates were mechanistically linked. Gyrase mutants with impaired activity caused the loss of from 30% to >95% of the diffusible supercoiling throughout most of the chromosome, while treatment with rifampicin that temporarily blocked transcription restored most of the lost supercoils in gyrase mutants. A gyrase defect also caused transcription elongation rates to decrease across the chromosome, and a mutation that reduced RNA polymerase efficiency increased average chromosome supercoiling levels. A model in which topoisomerases act close to highly transcribed operons to equilibrate the supercoil flux generated by transcription suggests that matched rates of gyrase turnover and transcription elongation speed determine the average supercoil density in bacterial chromosomes.

## Introduction

Negative supercoiling in bacterial DNA is generated by gyrase, which is composed of GyrA and GyrB proteins organized as A_2_B_2_ tetramers [1]. The average supercoil density of large bacterial chromosomes and small plasmid DNA is influenced by mutations in gyrase and two other topoisomerases. Topo I is a type Ia topoisomerase that breaks and rejoins DNA with a one-strand mechanism [2]. The enzyme is encoded by the essential gene *topA* [3] and it removes negative supercoils in a cofactor-independent reaction to protect chromosomes from toxic R-loops that can form at sites of high transcription [4]. Topo IV is a hetero-tetramer of ParC and ParE proteins in the form C_2_E_2_ [5]. With extensive homology to gyrase, Topo IV breaks both DNA strands simultaneously during the reaction cycle [2] and relaxes both positive and negative supercoils in steps of two supercoils per cycle in ATP-dependent reactions. Although Topo IV influences the supercoil density of chromosomal and plasmid DNA [6], its primary function is thought to be decatenation of sister chromosomes during final stages of chromosome segregation [7].

Changing the average supercoil density (σ) alters the efficiency and phenotype of many proteins involved in DNA replication [8], chromosome segregation [9]–[10], RNA transcription [11]–[13], homologous and site-specific recombination [14], and transposition [15]. Supercoil levels vary with growth conditions, and topoisomerase mutations arise as evolutionary adaptations in bacterial populations undergoing long-term growth on a monotonous carbon source [16]–[17]. Other than topoisomerases, our understanding of the roles of enzymes that contribute to the average supercoil density is poor, in part, because measuring supercoil density at specific locations of a 4 Mb chromosome is technically challenging.

**Figure 1:**
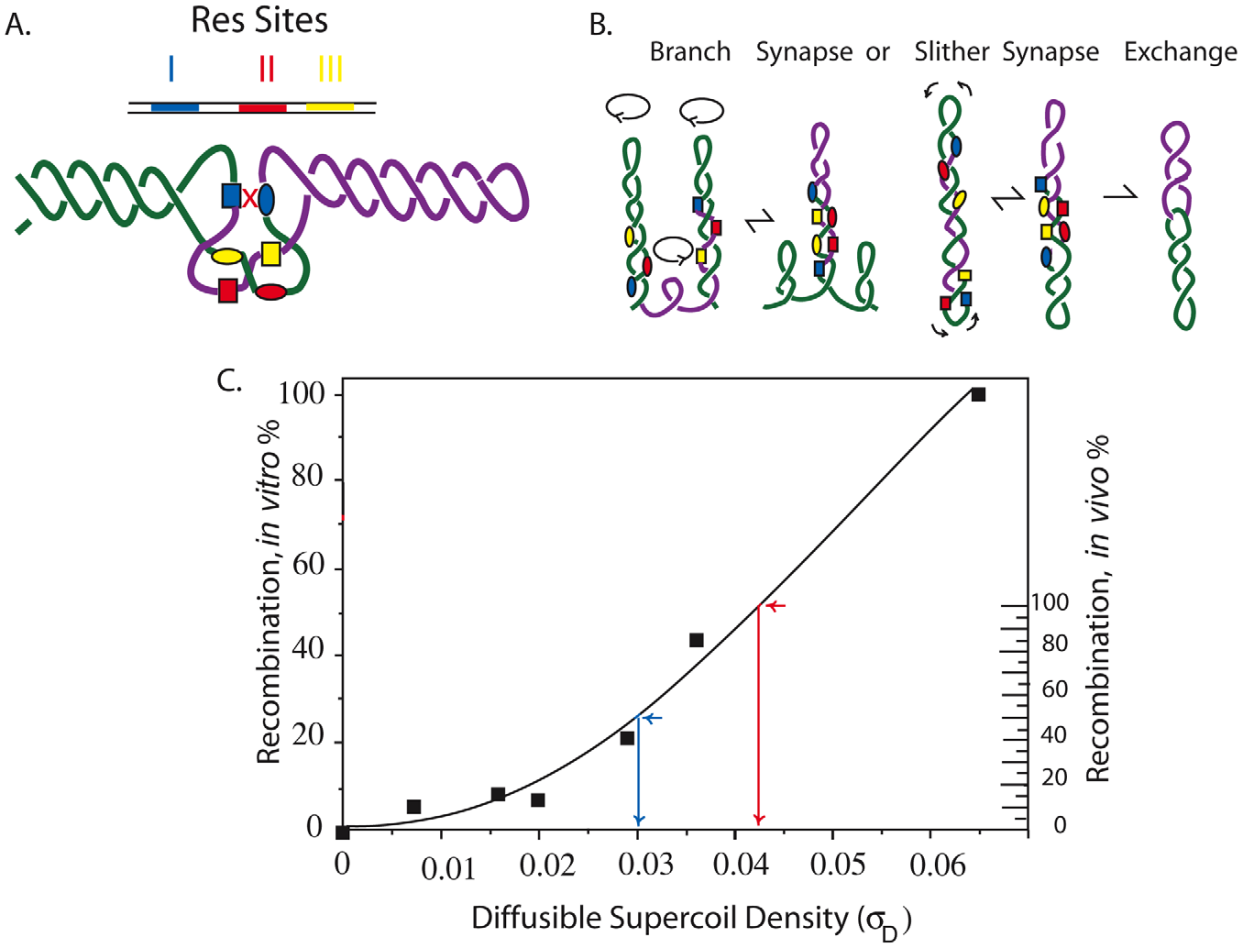
Mechanism of the γδ resolution reaction *in vitro* and *in vivo* showing how reaction efficiency correlates with (−) superhelix density. A. Recombination in the Tn3/γδ resolvase system requires a pair of 114 bp sites (Res) that include three binding sites for a dimer of the resolvase. The sites are I (blue), II (red), and III (yellow.) Supercoiling is required for the formation of a synapse in which two directly repeated Res sites entrap 3 negative crossing DNA nodes. Only resolvase dimers bound to Res site I, shown as blue boxes or blue ovals for different Res sites, can catalyze strand exchange. B. Movement of the interwound DNA strands promotes formation of the three-node tangle in A that occurs by reversible branching and slithering. Recombination results in an irreversible strand exchange that leaves two molecules linked as single supercoiled catenanes. C. The dependence of (−) supercoiling for plasmid recombination *in vitro* is shown by the scale on the left [22]. The inferred diffusible supercoil density for recombination of a 9 kb interval in the *Salmonella* chromosome *in vivo* is shown on the right [13].

Classical techniques used to measure chromosome supercoiling, like the ethidium bromide titration of nucleoids in sucrose gradients [18], give only an average supercoil density of the entire chromosome. The most common alternative method infers an average chromosomal supercoil density from the linking number of small plasmids in the same cell [19]. We developed techniques to monitor the supercoil-dependent movement of chromosomal DNA strands *in vivo* [8], [20]–[21]. The γδ site-specific recombination system uses supercoil diffusion to drive the assembly of a precise 3-node synapse of directly repeated Res sites (Figure 1A) [22]–[23]. Once a synapse forms, phosphodiester bond exchange leads to deletion of the intervening DNA segment without any accessory factors from *E. coli* [24]. The interwound DNA strands synapse by slithering and branching (Figure 1B). Slithering displaces two opposing strands along the axis of interwound loops. Branching rearranges the structure with new loops that grow and ebb laterally. If branching and slithering is unobstructed, resolution efficiency increases as the level of diffusible negative supercoiling increases, and deletions form rapidly and efficiently *in vitro* [25] and *in vivo* [26].

To analyze supercoiling at multiple locations, a 9 kb module called the “supercoil sensor” was developed [8]. It contains an entire Lac operon (*lacIZYA*) plus a selectable gentamycin resistance gene (Gn) flanked by directly repeated Res sites (Figure S1). The ends of the module are directly repeated Frt sites, which can be used to insert or extract sensors at unique chromosomal loci using the yeast 2 µ Flp recombinase (see Figure S1). The deletion efficiency of a LacI-repressed supercoil sensor is 50-fold more sensitive than a *gyrB*-*lacZ* promoter fusion, which varies by only 2-fold and has been used in many studies of chromosome topology in *E. coli* [12], [27]–[28].

The graph in Figure 1C illustrates the resolution response to negative supercoiling. Solid squares represent *in vitro* recombination rates (left Y axis) for endpoint assays carried out with plasmid DNAs with different supercoil densities (X axis). *In vivo*, about half of chromosomal σ is constrained (σ_C_) and half is diffusible (σ_D_) so that when σ = −0.060, σ_D_≥0.030. The scale on the right Y axis shows the resolution response to σ_D_ *in vivo*. Calculations of the apparent supercoil densities use the bottom half of the curve because after X-ray-induced relaxation of diffusible supercoiling, the *E. coli* chromosome retained a constrained supercoiling value of σ_D_ = 0.030 [29]. Resolution efficiency at σ_D_ = −0.030 is about 50% (blue arrow). When resolution efficiency approaches 100%, the σ_D_≥−0.040 (red arrow). We assume that *in vivo* reactions fall to 0 at σ_D_≤0.004.

Three regions near the *Salmonella* ribosomal *rrnG* operon have different supercoil properties during exponential growth in rich medium [13]. Recombination between γδ Res sites flanking the 5 kb *rrnG* operon was less than 1% because the presence of 60–80 RNA polymerases in the transcribed track blocked supercoil branching and slithering required for synapse. RNA polymerase unwinds a segment of the template strand at the active site, which represents −1.7 constrained supercoils per enzyme [30]; the accumulated supercoil density within the *rrnG* operon approaches σ_C_ = −0.290. When σ_C_ increased, σ_D_ decreased, but temporary interruption of RNA transcription by addition of rifampicin increased resolution efficiency 60 to 100-fold [13]. This result confirmed our earlier finding that highly transcribed genes are barriers to supercoil diffusion in the chromosome [31]–[32].

In 1987, Liu and Wang proposed that RNA polymerase generates two supercoiling domains during transcription [33]. The rationale was that rather than RNA polymerase rotating around DNA, the DNA duplex rotates (relative to the cytoplasm) due to the large inertial mass of polymerase, its associated transcription factors, and ribosomes that bind and translate the nascent mRNA during transcription elongation [34]. This model predicts a supercoil density difference with increased (−) supercoiling in DNA upstream and a loss of (−) supercoils downstream from expressed operons. We tested this model by placing a sensor upstream of the *rrnG* promoter and downstream of the transcription terminator. The upstream sensor had a 75% resolution efficiency compared to 28% for the downstream sensor [13], confirming the twin domain model and indicating a differential supercoiling value of Δ σ_D_ = +0.014 [13].

Previously, we measured what happens to twin domain supercoiling in strains with a mutant of Topo I (*topA217*) and TS *gyrA205* and *gyrB1820* mutants [13]. Each mutant caused the supercoil differential to increase in regions flanking the *rrnG* operon. In cells with the *topA217* mutation, upstream resolution efficiencies rose to 97% compared to 38% downstream. Conversely, a *gyrA205* mutant caused downstream resolution to fall to 9% compared to 60% resolution upstream. Most strikingly, a *gyrB1820* mutation caused downstream resolution to fall to 1% while recombination efficiency was 11% in the upstream domain. Local supercoiling levels were able to rise and fall dramatically at opposite ends of a highly transcribed operon in cells growing at permissive temperatures.

Here, we measured *Salmonella* chromosome supercoiling levels and transcription elongation rates using supercoil sensors at multiple positions covering the 6 macrodomains of *E. coli*. Our results show that rates of gyrase supercoiling and transcription elongation are linked. Temperature sensitive mutations in gyrase and Topo IV caused significant changes in genome-wide negative supercoil levels, even when cells were grown at a permissive temperature (30°). Transcription played a causal role in the supercoil losses because supercoiling rebounded after addition of rifampicin (Rif), which blocked transcription initiation. Our model is that transcription kinetics determine the optimal catalytic speed for gyrase, and the average chromosome supercoil density is an integral function of topoisomerases and RNA polymerase working in tempo together.

## Results

### Type II TS Topoisomerase Mutants of *Salmonella* Loose Supercoiling at 30°

During DNA synthesis, gyrase and Topo IV collaborate to remove (+) supercoils generated by fork movement [35]. However, their contribution to the dynamics of transcription has remained largely untested. We showed previously that some TS gyrase mutants cause a decline in (−) supercoiling at permissive growth temperatures in twin domains of the *rrnG* operon [13]. To study the general impact of transcription on chromosomal supercoil density, we evaluated 6 TS topoisomerase mutants for their influence on supercoil density near the origin of replication. Strains with TS alleles of gyrase and topo IV were constructed with a supercoil sensor placed between *gidB* and *atpI* (Figure S1). The Atp operon encodes a group of 9 highly expressed membrane proteins that generate ATP using the energy of the proton motive force across the cytoplasmic membrane. Each strain also carries the plasmid pJBRes 30′, which expresses a form of resolvase with a 30 min cell half-life.

All 4 subunits of gyrase and Topo IV were tested. GyrA contains the catalytic tyrosine residue that carries out DNA cleavage and re-ligation during the supercoiling reaction (Figure S2 A). *NH6016* carries the *gyrA213* ^TS^ allele (R358-H), which has a mutation located in the DNA-binding and cleavage domain [36]. Cultures were grown at 30° and doubling times were measured for each strain during mid log before resolution assays were carried out (Table 1 and Table 2). The complete derivation and genetic structure of each strain used in this manuscript is listed in Table S1.

**Table 1:** Resolution measurements and apparent σ_D_ values of WT, and TS mutants of gyrase (*gyrA, gyrB*) and Top IV (*parC, parE*).

Strain	Map Position	Relevant Mutation	Resolution Efficiency	MIF	Apparent σ_D_	Average σ_D_ (w/o Cs 33)
*NH3868*	Cs 57.65	None	75±6%	1	−0.037	
*NH4028*	Cs 57.64	None	28±3%	1	−0.023	
*NH6000*	Cs 85	None	81±4%	1	−0.038	
*NH6001*	Cs 71	None	82±2%	1	−0.038	
*NH6002*	Cs 58	None	80±3%	1	−0.038	
*NH6003*	Cs 45	None	73±6%	1	−0.036	
*NH6005*	Cs 33	None	45±6%	1	−0.022	
*NH6006*	Cs 21	None	92±2%	1	−0.041	
*NH6007*	Cs 9	None	73±12%	1	−0.036	*WT*
*NH6008*	Cs 96	None	85±3%	1	−0.040	−0.036±.005
*NH6016*	Cs 85	*gyrA213*	58±1%	1.4	−0.032	
*NH6019*	Cs 85	*gyrA209*	30±12%	3	−0.024	
*NH6020*	Cs 71	*gyrA209*	30±6%	3	−0.024	
*NH6021*	Cs 58	*gyrA209*	26±9%	3	−0.022	
*NH6022*	Cs 45	*gyrA209*	17±5%	5	−0.018	
*NH6024*	Cs 33	*gyrA209*	9±4%	5	−0.013	
*NH6025*	Cs 21	*gyrA209*	20±13%	5	−0.019	
*NH6026*	Cs 9	*gyrA209*	30±3%	2	−0.02	*gyrA209*
*NH6027*	Cs 96	*gyrA209*	48±5%	2	−0.030	−0.023±.004
*NH6028*	Cs 85	*gyrB652*	7±3%	12	−0.012	
*NH6029*	Cs 71	*gyrB652*	<1%	>80	<−0.003	
*NH6030*	Cs 58	*gyrB652*	2±1%	40	−0.004	
*NH6031*	Cs 45	*gyrB652*	<1%	>70	<−0.003	
*NH6033*	Cs 33	*gyrB652*	<1%	>45	<−0.003	
*NH6034*	Cs 21	*gyrB652*	<1%	>90	<−0.003	
*NH6035*	Cs 9	*gyrB652*	<1%	>70	<−0.003	*gyrB652*
*NH6036*	Cs 96	*gyrB652*	3±1%	28	−0.007	−0.005±.003
*NH6040*	Cs 85	*parC281*	76±1%	1.1	−0.037	
*NH6043*	Cs 85	*parE206*	59±4%	1.4	−0.033	
*NH6044*	Cs 71	*parE206*	66±4%	1.2	−0.034	
*NH6045*	Cs 58	*parE206*	64±3%	1.1	−0.034	
*NH6046*	Cs 45	*parE206*	60±6%	0.8	−0.032	
*NH6048*	Cs 33	*parE206*	27±2%	1.7	−0.021	
*NH6049*	Cs 21	*parE206*	58±3%	1.6	−0.032	
*NH6056*	Cs 9	*parE206*	51±4%	1.4	−0.030	*parE206*
*NH6058*	Cs 96	*parE206*	63±4%	1.3	−0.033	−0.032±.001

**Table 2:** TS alleles of gyrase and Topo IV decrease diffusible chromosome supercoiling at Cs 85.

Strain Number	Relevant Genotype	Doubling Time (min)	Resolution Efficiency	Apparent σ_D_	MIF
*NH6000*	WT	39±1	81±3%	−0.038	1
*NH6016*	*gyrA213* TS	39±1	58±1%	−0.032	1.4
*NH6019*	*gyrA209* TS	45±3	30±13%	−0.026	2.7
*NH6028*	*gyrB652* TS	53±3	7±3%	−0.010	12
*NH6037*	*gyrB1820* TS	58±4	8±6%	−0.011	10
*NH6040*	*parC281* TS	39±1	76±1%	−0.037	1.1
*NH6043*	*parE206* TS	52±2	59±4%	−0.032	1.4

Resolution assays for a Lac-Gn module introduced at Cs 85 were measured in WT and TS mutations of gyrase and Topo IV. Two mutations in gyrA and gyrB genes plus 1 TS allele of the *parC* and *parE* genes of Topo IV were tested in exponential phase at 30°. The apparent σ_D_ was estimated from the graph in Figure 1C. The MIF is calculated as resolution efficiency of the WT divided by the mutant and it indicates the magnitude effects of each allele on recombination at Cs 85.

Strain NH6016 had the same doubling time as WT (39±1 min) but the resolution efficiency fell from 81±3% for WT to 58±1%, representing a 28% loss in recombination efficiency. To compare alleles, we define a term Mutant Impact Factor (MIF) to be the resolution efficiency of the WT strain divided by the resolution efficiency of an isogenic mutant. A large MIF indicates a dramatic change in supercoiling. *NH6016* had a significant MIF of 1.4. A *gyrA209* ^TS^ allele (G597-D) in *NH6019* alters the second ß-propeller of GyrA, which contributes to DNA-looping that forms a chiral (+) node [37]. The *gyrA209* doubling time increased from 39±1 min to 45±3 min, which is a 15% decrease in growth rate. The resolution efficiency in this strain fell to 30±12%, resulting in a MIF of 2.7. Thus, two *gyrA* ^TS^ mutants had reduced resolution efficiency, which indicates a loss of (−) supercoiling even at the permissive temperature of 30°.

The GyrB subunit encodes the ATP-binding domain of gyrase, which couples ATP binding to a large conformation shift that drives negative supercoiling reactions (Figure S2 B). Two GyrB mutants were tested. *NH6028* contains the *gyr652* mutation (R436-S), which alters a Mg^++^ binding domain that coordinates structural conformation changes during DNA cleavage [38]. This enzyme has a low k_cat_ relative to WT gyrase [8], and the doubling time of strains with this mutation increased by 36% to 53±3 min. The resolution rate in *NH6028* was 7±3% (Table 2), and the resulting MIF of 12 indicates dramatic loss of supercoil density. The scale in Figure 1 predicts a change of +0.027 in the supercoil sensor for an apparent σ_D_≤−0.012. A second allele, *gyrB1820* ^TS^ (C56-Y) alters the ATP binding domain that dimerizes and then hydrolyzes two ATPs during the catalytic cycle [39]. This mutant is the most severe allele in our *Salmonella* gyrase collection. The doubling time at 30° increased by almost 50% to 58±4 min. *NH6037* had a resolution efficiency of 8±6%, resulting in a MIF of 10. Thus, the supercoil sensor shows that two TS *gyrA* and two TS *gyrB* alleles cause significant losses in (−) chromosome supercoiling at the ATP operon location.

### Topo IV TS Mutants Lose Negative Supercoiling

Topo IV has a subunit structure, catalytic mechanism, and sensitivity to drugs novobiocin and fluoroquinolones that is similar to gyrase [28]. Although its primary function is decatenation and untangling of sister chromosomes prior to segregation and cell division [7], Topo IV relaxes both (−) and (+) supercoils *in vitro* and contributes to the dissipation of (+) supercoils during DNA replication *in vivo* [35]. Therefore, we tested the impact of TS Topo IV alleles on chromosomal supercoiling. The ParC subunit catalyzes DNA breakage/reunion during strand passage reactions, and *NH6040* has the *parC281* ^TS^ (P556-L) mutation, which resides in a region with no known function. This mutant showed no difference in growth rate from the WT (39±1 min) and resolution efficiency was 76±1%, which is close to the WT (81±4%) with a MIF of 1.1. ParE functions like GyrB, binding and hydrolyzing ATP to fuel cycles of strand transfer. The *parE206* (V67-M) mutation in strain *NH6043* is in the ATP binding domain of Topo IV, and the doubling time at 30° increased by 33% to 52±2 min. The resolution efficiency was 59±4% (Table 1), yielding a MIF of 1.4. Therefore, the defect in the ParE206 subunit of Topo IV caused a supercoil loss comparable to GyrA213.

### WT Supercoiling Levels Are Similar in 5 Macrodomains

The *E. coli* chromosome appears to have multiple levels of organization. In addition to 10 kb domains that restrict supercoil diffusion [40], a long range order called macrodomains has been proposed [41]. Macrodomains represent segments of 0.6 to 1 Mb that may coalesce in the folded chromosome. The first indication of macrodomain structure came from fluorescent in situ hybridization (FISH) with the Ori and Ter regions occupying distinct positions near the opposing cell poles in newborn cells [42]. The Boccard laboratory extended the *E. coli* framework to include three additional segments and two less structured regions by measuring the interaction frequencies of pairs of λ attachment sites distributed across the chromosome [41], [43]–[44]. Although the efficiency of λ site-specific recombination shows variation at specific points in *Salmonella* [45], the macrodomains proposed for *E. coli* may or may not be conserved along with gene order that is shared between these species.

**Figure 2:**
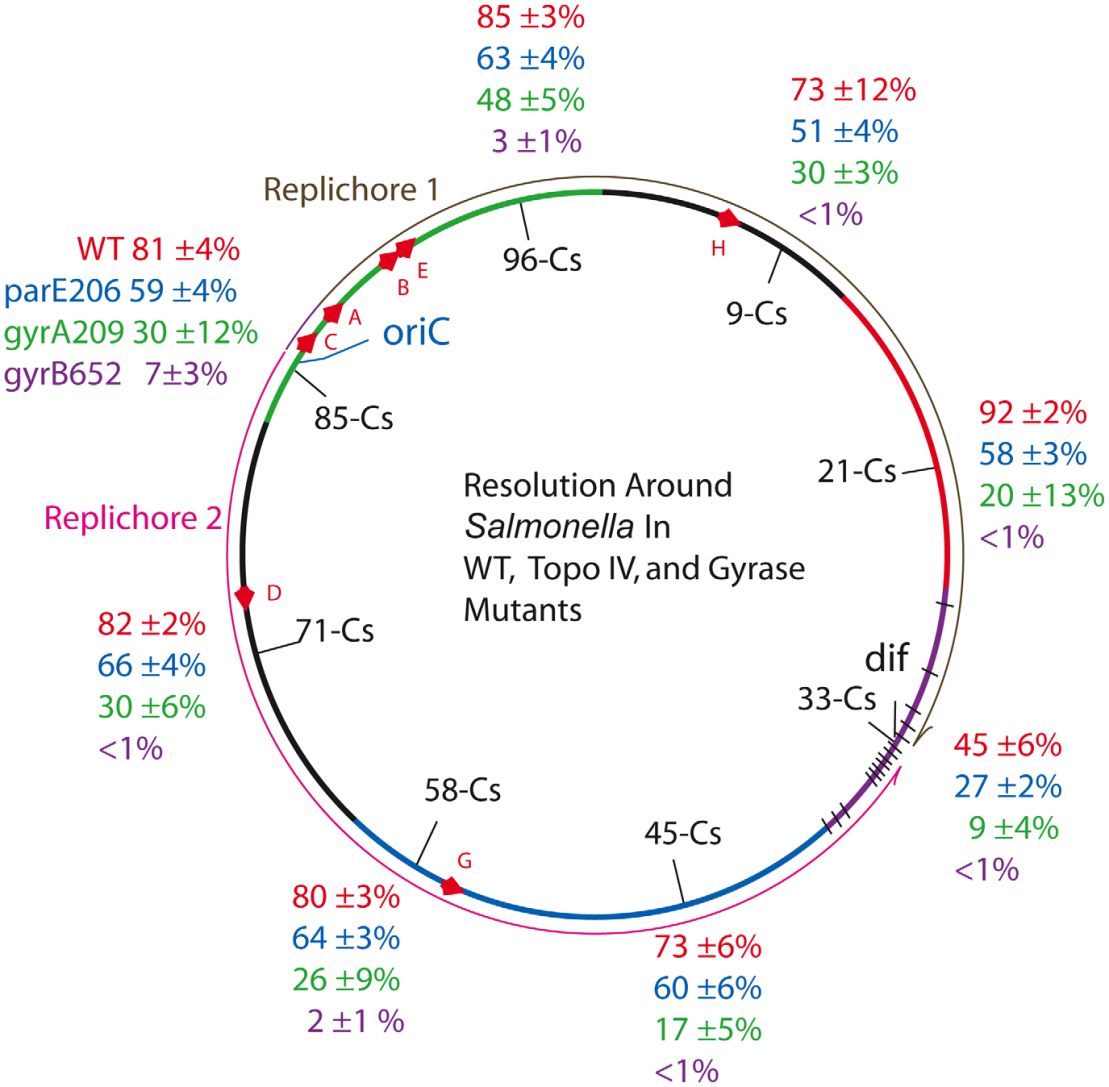
Resolution efficiencies in the *Salmonella* chromosome decline in strains carrying TS mutations in gyrase and Topo IV, even when cells are grown at permissive temperature (30°). Recombination reactions at 8 locations around the *Salmonella* chromosome was studied in 32 strains described in Table 1. The experiment covers the 6 macrodomains of *E. coli*, shown as color coded arcs superimposed on the *Salmonella* map: green, Ori domain; black, Right Unstructured domain; red, Right domain; purple, Ter domain with black hatches showing *matS* sites; blue, Left domain; and black, Left Unstructured domain [41]. The direction of replication fork movement in replichore 1 (brown) or 2 (pink) is shown by arrows outside the circle. Each strain had a 9 kb Lac-Gn supercoil sensor inserted between consecutive genes, plus a plasmid that contains a thermo-inducible γδ resolvase with a 30 min half life (Materials and Methods). Recombination data and estimated values of apparent diffusible supercoiling for each experiment are reported in Table 1.

Supercoil levels in all potential macrodomains were measured by introducing sensors into 7 more sites in *Salmonella* to include at least one measurement in each *E. coli* macrodomain (Figure 2). *E. coli* chromosome map coordinates are notated in minutes that reflect the HFR transfer time of each genetic region during a standard mating experiment (1–100 min). The *Salmonella* chromosome has a gene order that is highly congruent with *E. coli*, but with numerous inserted gene islands, the genome size is 5% larger. To compensate, map coordinates in *Salmonella* are described in units of 100 centisomes (Cs) with the same starting position as in *E. coli*. The largest *E. coli* macrodomain is Ori, which spans 930 kb of DNA. The corresponding segment in *Salmonella* extends from Cs 81 to Cs 1 in Figure 2 (the green arc). Ori includes 4 of the 7 ribosomal RNA operons and many highly transcribed genes involved in transcription and translation. 70% of the RNA polymerase in rapidly dividing cells is confined to this chromosome sector. The module at Cs 85 (Table 1) is near the left edge of the Ori macrodomain in replichore 2; it had a recombination efficiency of 81±4%. In *NH6008*, resolution efficiency was tested in another segment of the Ori domain in replichore 1. The sensor disrupts the *Salmonella* gene *STM4442*, which encodes a small putative “cytoplasmic protein” at Cs 96. *NH6008* matched *NH6000* with a resolution efficiency of 85±3% (Table 1).

Two domains reside exclusively in replichore 1. The Right Unstructured region is shown in black (Figure 2) clockwise of *oriC*. The smallest macrodomain in *E. coli* (560 kb), it extends from Cs 1 to Cs 13 in *Salmonella*. A sensor was inserted at Cs 9 in *NH6007* between *ampH*, which encodes a beta-lactam binding protein, and *sbmA*, a gene encoding an inner membrane ABC transporter. *NH6007* had a resolution efficiency of 73±12%. The Right macrodomain of *E. coli* spans 600 kb from Cs 13 to Cs 26. In *NH6006*, a sensor was inserted at Cs 21. This is the only position in which a reporter lies between two divergently transcribed genes. These genes are *STM0951*, which encodes a “cytoplasmic protein” transcribed in the counterclockwise direction, and *STM0952*, which is a transcription regulatory protein transcribed in the clockwise direction. The recombination efficiency in *NH6006* was the highest measured at 92±2%.

Two *E. coli* macrodomains reside entirely in replichore 2. The Left Unstructured region is a 550 kb sector. The comparable region of *Salmonella* is shown in Figure 2 as a black arc counterclockwise of *oriC* running from Cs 81 to Cs 62. A sensor inserted at Cs 71 lies between *STM3261*, which encodes a galacticol-1-phosphate dehydrogenase, and *STM3262*, a putative repressor in strain *NH6001*. The resolution efficiency was 82±2%. (Table 2, Figure 2). The Left macrodomain in *E. coli* is an 892 kb region extending from Cs 62 to Cs 43, shown as a blue arc. Two modules were placed in this segment of *Salmonella*. In *NH6002*, a module resides at Cs 58 between *smpB*, which makes a small protein that may bind the SsrA subunit of the SsrA/SsrB two-component regulatory complex [46], and pseudogene *STM2689*. A second module in this sector is integrated between *STM2135*, which encodes an inner membrane protein, and the protease-encoding gene *yegQ* at Cs 45. The deletion efficiencies of *NH6002* and *NH6003* were 80±3% and 73±6%, respectively.

The macrodomain that lies across from Ori in *E. coli* is the Ter domain (purple arc), which is a 780 kb region of *E. coli*. Ter has 24 copies of a unique 14 bp site called *matS* that is found uniquely in this segment. The *matS* sites bind MatP, which may organize them into a single focus in cells with a chromosomal MatP-GFP fusion. One model is that 23 Ter domain loops are formed with a central hub of MatP protein [47]. In *Salmonella*, the Ter domain may be a smaller 560 kb region with only 14 predicted *matS* sites [47] (black lines in Figure 2). In *NH6005*, a sensor was inserted at Cs 33 between the pseudogene *STM1553* and STM1554, which encodes a putative “coiled coil protein.” The resolution efficiency here was lower than any other site tested in the survey, 45±6%.

The cumulative average resolution efficiency of sensors located at 7 regions (excluding the Ter domain) was 81±7% and the apparent σ_D_ = −0.038±.002. There was no statistically significant variation in supercoil levels from the Ori to the terminus. At Cs 33, the resolution efficiency of 45% is roughly half that measured at the other 7 sites. At this location, a Res site is only 470 bp from *dif*. We believe that resolvase binding to the site nearest *dif* may be occluded by DNA-binding proteins unique to the region. These proteins include *matS*-MatP complexes [47], the FtsK DNA translocation motor complex [48], a high affinity site for Topo IV [49], and XerC/D proteins that bind *dif* to catalyze complex topological reactions that untangle and separate sister chromosomes [50].

### TS Alleles of Gyrase and Topo IV Cause a General Loss of Supercoiling

With the exception of the Ter macrodomain, the genes and mechanisms that organize the Ori, Right, and Left macrodomains are undefined. Supercoiling is an important factor in bacterial DNA condensation, so we tested the impact of topoisomerase mutations in all domains using the supercoil sensor. In the strain series *NH6019*-*NH6027*, each strain has the *gyrA209^TS^* allele, which showed a MIF of 2.7 at the Cs 85 position (Table 2). The resolution efficiency measured at 7 positions (excluding position Cs 33) showed more variability than the WT set (Table 2 and Figure 2, green characters). The average recombination efficiency was 28±9%. This drop corresponds to a mean MIF of 3. The estimated change in σ_D_ relative to WT at 7 positions was +0.013. Previous work from other laboratories showed that growth of *E. coli* cells stopped when supercoiling dropped to this level [3], [51]–[52].

Supercoil losses at 7 locations in *gyrB652* ^TS^ mutants were larger than those measured at Cs 85 (Table 2). The resolution efficiency at Cs 71 – *NH6029*, Cs 45 – *NH6031*, Cs 33 – *NH6033*, Cs 21 – *NH6034*, and Cs 9 – *NH6035* were all less than 1% (Figure 2, purple characters). The estimated value of σ_D_ dropped from −0.038 to an apparent σ_D_ = <−0.004. Resolution efficiency was near the detection limit at Cs 58 – NH6030 (2±1%) and at Cs 96 – *NH6036* (3±1%). Averaging across 7 points on the chromosome, the mean recombination efficiency was 2±2% and the MIF was 40. Surprisingly, this strain with a greatly relaxed chromosome has a doubling time only 36% longer than WT (53±3 vs. 39±1).

To see if Topo IV has a related genome-wide supercoil phenotype, the *parE206* ^TS^ allele of Topo IV was tested (Figure 2, blue characters). The resolution efficiency at Cs 85 (59±4%) was similar to results at positions Cs 71, 66±4%; Cs 58, 64±3%; Cs 45, 60±6%; Cs 21, 58±3%; Cs 9, 51±4%; and Cs 96, 63±4% (Figure 2, blue characters). The mean recombination efficiency at 7 chromosomal positions fell to 60±5% for a MIF of 1.4. Again, the Ter macrodomain at Cs 33 showed lower resolution efficiency than all other locations. *NH6048* recombined at 27±2% compared to 45±6% in WT.

### Transcription Causes Supercoiling Losses in Gyrase Mutants

Replication and transcription generate positive supercoils in regions downstream of replisomes and highly expressed operons, respectively. To understand the reason a TS GyrB mutant loses most of the detectable diffusible chromosomal supercoiling, we tested the role of transcription. Like E. coli, WT *Salmonella* is organized into 400–500 domains that limit supercoil diffusion [21]. Topo I relaxes negative supercoils generated upstream of highly transcribed regions. If gyrase can't supercoil DNA at rates matching the rotation speeds downstream of the 7 ribosomal RNA operons, the multiple tRNA genes, and 30 highly transcribed protein-encoding genes that are spread out over the chromosome, then transcription could run down reservoirs of stored supercoils in low transcribed regions. Supercoil depletion might also be a consequence of having all highly transcribed genes oriented in the same direction as replication, presumably to mitigate effects of head on replisome-RNAP collisions [53].

**Figure 3:**
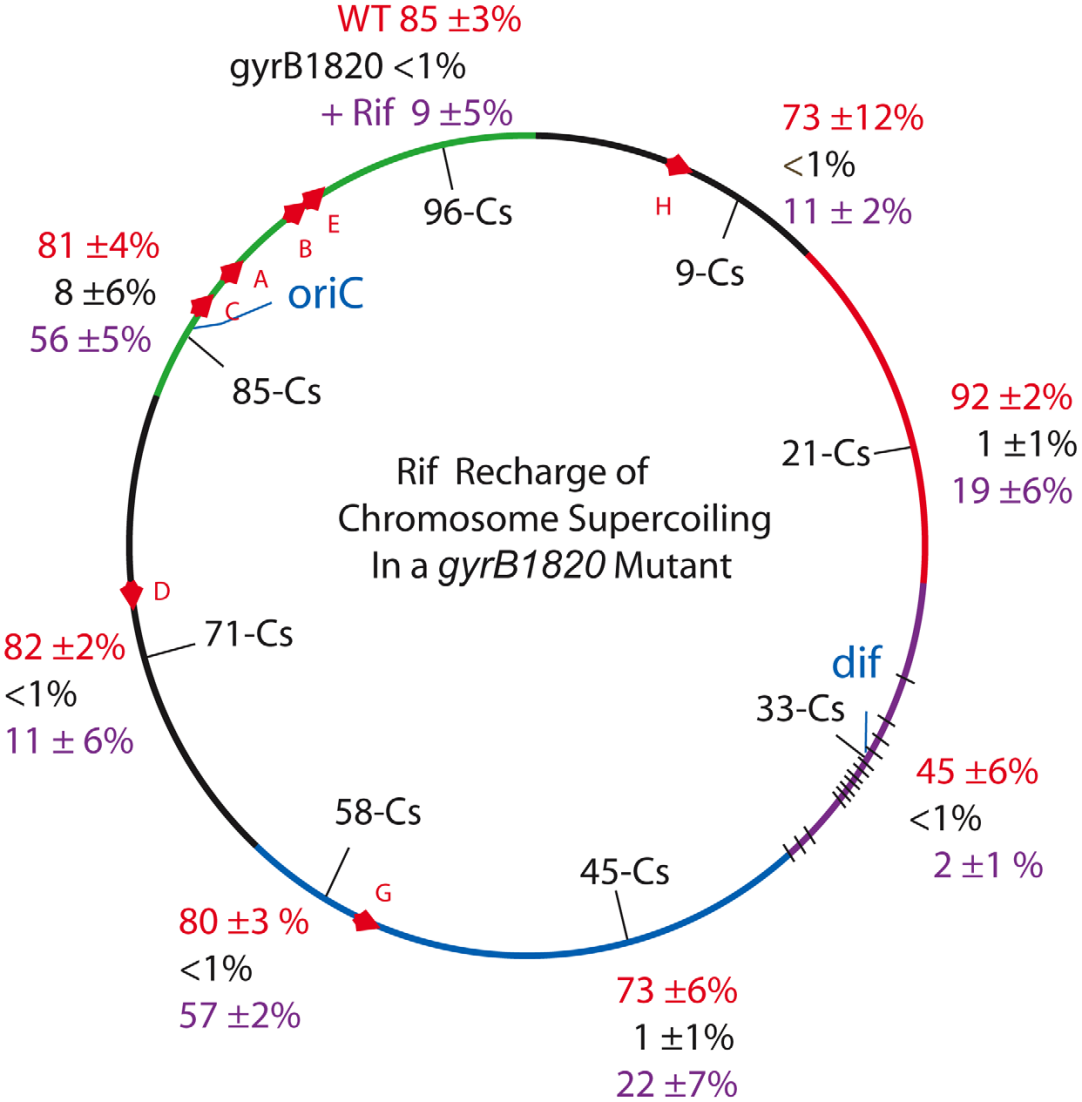
Interrupting transcription causes a dramatic rebound in resolution for strains carrying the GyrB1820 gyrase. Recombination efficiencies of supercoil sensors at 8 positions are shown for WT (red) and *gyrB1820* ^TS^ mutants tested without Rif (black). The purple numbers show recombination rates after rifampicin was added to cultures immediately following the 10 min induction of resolvase and rifampicin was subsequently washed out of cells 30 min later.

To test the role of transcription in supercoil regulation, a strain set carrying the severe *gyrB1820* ^TS^ mutation was constructed (Table 3, *NH6037*-*NH6114*). Similar to cultures with the *gyrB65*2 mutation, the resolution efficiency was at the detection limit (≥1%) at all locations other than Cs 85 (Table 3, Figure 3, black numbers). The average recombination value at 7 sites was 1.6±3% (entering values of 0.5% for measurements <1%) and the MIF mean was 50.

**Table 3:** Rifampicin induced recovery of chromosomal σ_D_ in *gyrB1820* mutants.

Strain	Map Position	Relevant Mutation	Resolution Efficiency	MIF	Efficiency +Rif	Increased σ_D_+Rif
*NH6037*	Cs 85	*gyrB1820* ^TS^	8±6%	9	56±5%	−0.020
*NH6108*	Cs 71	*gyrB1820* ^TS^	<1%	>80	11±6%	−0.012
*NH6109*	Cs 58	*gyrB1820* ^TS^	<1%	>80	57±2%	−0.030
*NH6110*	Cs 45	*gyrB1820* ^TS^	1±1%	>73	22±7%	−0.018
*NH6111*	Cs 33	*gyrB1820* ^TS^	<1%	>45	2±1%	−0.004
*NH6112*	Cs 21	*gyrB1820* ^TS^	1±1%	90	19±6%	−0.016
*NH6113*	Cs 9	*gyrB1820* ^TS^	<1%	>73	11±2%	−0.012
*NH6114*	Cs 96	*gyrB1820* ^TS^	<1%	>85	9±5%	−0.011

Resolution assays were carried out as described in Materials and Methods. Each result is the product of 3 independent replicas ±1 SD of the mean. The value of σ_D_ was estimated from the plot in Figure 1C.

**Figure 4:**
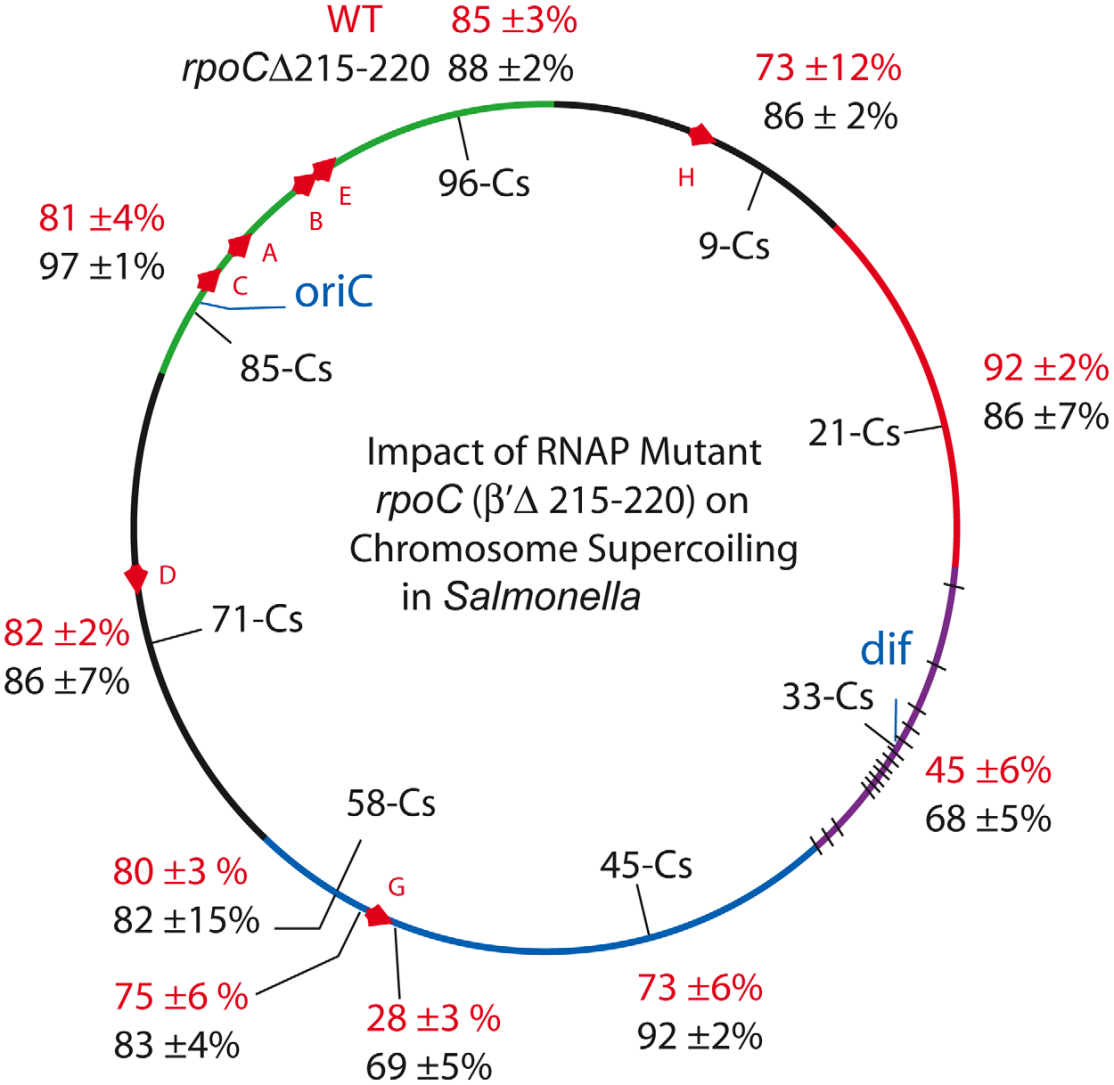
An RpoC mutant that slows transcription and mimics the stringent response in the absence of ppGpp causes global increases in resolution efficiency in the *Salmonella* chromosome. Resolution assays for Lac-Gn modules around the *Salmonella* chromosome are shown for WT (red) and the *rpoC* mutant (black).

We added Rif to aliquots of each culture immediately after the 10 min resolvase induction period. Rif blocks transcription initiation, but elongation and termination occurs normally; no cell death was associated with drug treatment. After 30 min of incubation, the drug was washed out and the recombination efficiencies were measured after cells doubled more than twice, to allow chromosome segregation. Rif had a dramatic impact on resolution efficiency (Table 3, Figure 3, black numbers). At Cs 85 - *NH6037*, resolution was 8±6% with a MIF of 10. Rif treatment increased resolution 7-fold to 56±5% and the MIF dropped to 1.4 (Table 3 and Figure 3, purple numbers). Dramatic results were also observed at 6 other locations. In strains with modules at Cs 9, Cs 71, and Cs 96, resolution rose at least 10-fold from ≤1% to 11±2%, 11±6%, and 9±5% respectively (Figure 4, Table 3). At Cs 45, resolution increased from <1% to 22±7%. The largest improvement was observed in *NH6109* at Cs 58 where resolution increased >60 fold from <1% to 57±2%. Like the *gyrB652* ^TS^ strain set (Figure 2), resolution efficiency in the Ter domain at Cs 33 was low and remained low after Rif addition, rising only from <1% to 2±1%. Overall, excluding the Ter domain, Rif addition reduced the MIF mean from 50 to 3.

### Supercoil Density Increases in Strains with a Mutant RNA Polymerase

The increase in resolution after Rif addition supports the hypothesis that general transcription can deplete supercoil levels when gyrase is impaired. But what would happen to supercoiling in strains with a WT complement of topoisomerases and a slow RNA polymerase? If catalytic rates of transcription and supercoiling are under selection to match, would such cells experience a general supercoil increase? Deletion of 6 amino acids in the ß′ subunit (RpoC Δ Δ215–220) makes a form of RNA polymerase with a constitutive low transcription rate for stable RNA, including the 7 ribosomal RNA operons [54]. This mutation was introduced to *Salmonella* strain set (*NH6206*-*NH6215*), which included sensors upstream and downstream of *rrnG*, increasing the number of test locations to 10 (Table 4). The doubling time of the mutant growing at 30° increased by 28% from 39±1 min in WT to 50±2 min. Remarkably, the resolution efficiency increased throughout the mutant chromosome, except for one position at Cs 21, which was within experimental error of matching the highest efficiency in the WT RNA polymerase strain (92±2 - 86±7, Table 4, Figure 4). The WT mean resolution efficiency at 10 positions was 74±18%, whereas the RpocΔ215–220 average was 85±8% with a MIF of 0.87. A 13% increase in resolution represents an apparent mean change of Δσ_D_ = −0.004. Interestingly, the impact of the *rpoC* mutation was greatest at positions where the WT resolution levels were lowest. For sensors adjacent to the *rrnG* operon at Cs 57.64 and Cs 57.65, the upstream sensor increased from 75±6% resolution to 83±4% and the downstream location changed from 28±3% to 69±5% resolution. The downstream location had a MIF of 0.41, proving that locations where gyrase worked the hardest benefited the most from reduced transcription rates.

**Table 4:** Impact of a 6 amino acid *rpoC* deletion on *Salmonella* resolution efficiency.

Strain	Map Position	Relevant Mutation	Resolution Efficiency	MIF
*NH6206*	Cs 85	*rpoC* Δ215–220	97±1%	0.83
*NH6207*	Cs 71	*rpoC* Δ215–220	86±7%	0.95
*NH6208*	Cs 58	*rpoC* Δ215–220	82±15%	0.98
*NH6209*	Cs 45	*rpoC* Δ215–220	92±2%	0.79
*NH6210*	Cs 33	*rpoC* Δ215–220	68±5%	0.66
*NH6211*	Cs 21	*rpoC* Δ215–220	86±7%	1.07
*NH6212*	Cs 9	*rpoC* Δ215–220	86±2%	0.84
*NH6213*	Cs 96	*rpoC* Δ215–220	88±2%	0.96
*NH6214*	Cs 57.65	*rpoC* Δ215–220	83±4%	0.90*
*NH6215*	Cs 57.64	*rpoC* Δ215–220	69±5%	0.41*

Resolution assays were done as described in Materials and Methods.

* The MIF in NH6214 and NH6215 was calculated from WT results upstream and downstream of the *rrnG* operon in Booker et al [13].

### A GyrB1820 Mutation Decreases RNAP Elongation Rates

**Figure 5:**
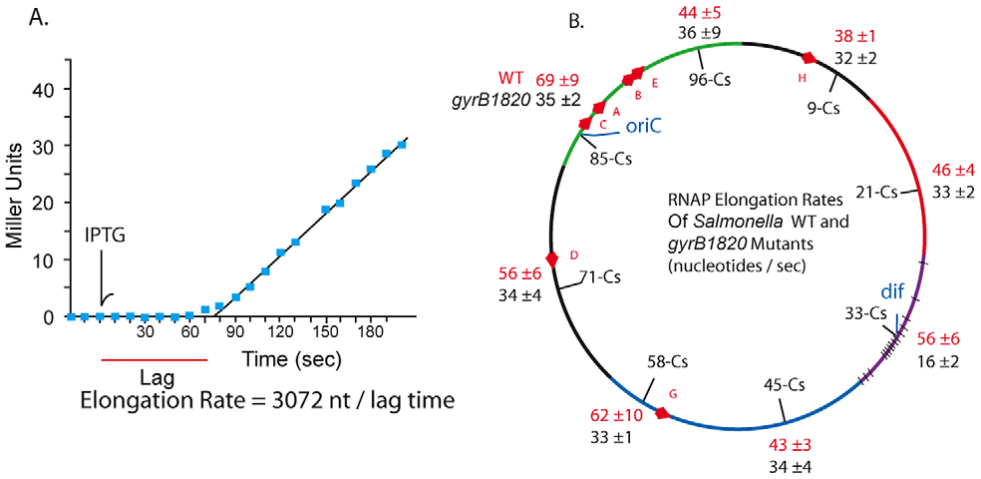
RNAP elongation rates at 8 chromosomal loci in WT (red) and *gyrB1820* mutant strains (black) are significantly reduced by the GyrB1820 mutation. A. 20 ml cultures growing in minimal (AB) medium with glucose were grown at 37° to an OD A_600_ = 0.20. Three 0.5 ml aliquots were taken, added to ice-cold ZS buffer and saved for a base line reading. IPTG was added to a concentration 1.5 mM at the time point indicated by the arrow, and samples were removed at 10 sec intervals. The chromogenic substrate ONPG was added to each culture in timed assays that extended for 1.5–3 h, depending on the activity level. B. The mRNA elongation rate was calculated by dividing the 3072 nt *lacZ* mRNA by the lag time to linear increase in β-Gal, giving the rate in units of mRNA nt/sec.

In 1973 Pato, Bennett, and von Meyenberg discovered that the rates of transcription elongation and translation were closely matched for most genes in *E. coli* [34]. Could the transcription rate include a role for gyrase? We measured the coupled *lacZ* transcription/translation kinetics at 8 locations in WT and *gyrB1820* mutants. The method is outlined in Figure 5 A. Cultures grown in minimal medium plus glucose were sampled at 10 sec intervals and placed on ice in lysis buffer [55]. The first three samples established a baseline, then IPTG was added to each culture at a final concentration of 1.5 mM, and 10 sec sampling was continued. After all samples were collected, the chromogenic substrate ONPG was added to timed reactions that ran at 37° for 1.5 to 3 h. The transcription rate in nucleotides per second (nt/sec) is calculated as the length of the LacZ transcript (3072 nt) divided by the lag time to the start of a linear increase in enzyme activity (Figure 5A). Each strain was tested in triplicate using different colonies, and the transcription rates with one standard deviation are shown for WT (red) and GyrB1820 mutants (black) in Figure 5B.

Unexpectedly, coupled transcription/translation rates varied at different positions in the *Salmonella* genome. The fastest transcription speeds were 69±9 nt/sec at Cs 85 and 62±10 nt/sec at Cs 58. These sites were 45% faster than the 38±1 nt/sec rate measured at Cs 9. The average elongation rate in WT cells across all positions was 52±10 nt/sec. The impact of a *gyrB1820^TS^* mutation was tested in strain set NH6222-NH6229. Elongation at 7 positions fell to a uniform mean of 32±6 nt/sec, which is 40% slower than the average of these positions in WT. Again, the Ter domain at Cs 33 was different. Transcription/translation rates at *dif* fell from 56±6 nt/sec to 16±2 nt/sec in *gyrB1820*. These results together with the experiments using Rif suggest to us that unique factors influence resolution efficiency and transcription near *dif*. Nonetheless, throughout most of the genome, and in at least 5 macrodomains, transcription/translation rates and gyrase supercoiling efficiency were covariant.

## Discussion

### Transcription Contributes to Supercoil Regulation

Three results show that the mean supercoil density of *Salmonella* DNA is determined by a mechanism that links the catalytic efficiency of gyrase to the elongation rate of transcription. First, TS alleles of GyrB caused a broad and dramatic depletion of (−) supercoiling throughout the *Salmonella* genome (Figure 2). This effect was largely reversed by temporarily blocking transcription with Rif (Figure 3). Second, supercoil densities rose above the WT level in cells carrying a mutant ß′ subunit (RpocD215–220) (Figure 4). Third, the GyrB1820 mutation caused the rates of coupled LacZ transcription/translation to decrease from the WT mean of 52±10 to 32±6 nt/sec over most of the genome (Figure 5).

The impact of TS mutations in both GyrA and GyrB on resolution efficiencies for cells growing exponentially at a permissive temperature of 30° was unexpected (Table 1, Table 2, and Figure 2). There are three plausible explanations for this reduction in recombination rates: 1) When the catalytic rate of gyrase was slowed by mutation, the loss of negative supercoiling downstream of highly transcribed genes was spread across the genome. 2) The slow growth rate in gyrase mutants caused a drop in resolvase expression that limited recombination. 3) A slow growth rate induced increased expression or rearrangement of nucleoid-associated-proteins (NAPs) that constrained (−) supercoiling [56]–[58] and/or occluded resolvase binding to Res sites.

A change in the resolvase expression level does not explain the γδ recombination results for two reasons. First, we analyzed resolvase in WT and mutant strains using Western blots. The resolvase band at 21 KDa appeared after thermo-induction in all strains tested (Figure S3.) The expressed resolvase contains an SsrA degradation tag appended as the terminal 11 amino acids, and this tag limited the *in vivo* protein half-life to under 30 min [21]. Resolvase disappeared during a 30 min incubation at 30° following the 42° incubation, including the cells treated with Rif [21]. Whereas the resolvase band intensity varied somewhat between different strains, the band variation did not correlate with the ratios of WT to mutant catalytic resolution efficiency. These results agree with our earlier finding that a 5–10 fold decrease in resolvase expression seen in stationary phase cells does not limit resolution [20].

Second, a much more compelling argument comes from the Rif experiment shown in Figure 3. When transcription was unobstructed, the resolution efficiencies in *gyrB1820* strains were at the detection limit of 1% at 7of 8 genome locations. But resolution increased at Cs 85 and Cs 58 to 56% when Rif was added to the cultures after downshift to 30°. An interruption in transcription restored 70% of the resolution efficiency at these two sites compared to that seen in WT cells. The length of time that cells were exposed to resolvase was previously shown to be a factor in resolution efficiency [21], and after transcription inhibition, resolvase exposure dropped from about 40 min (induction plus incubation time) to 25 min or less. Yet, at 7 chromosome locations covering 5 macrodomains (excluding Ter), the mean resolution efficiency for GyrB1820 without Rif was 1.6±3% with a MIF = 50 (81%/1.6%), and after Rif, the mean efficiency rose to 26%±21% with the MIF shrinking to 3 (81%/26%). The variation in resolution around the chromosome after the addition of Rif could mean that extra factors contributed (i.e. increased constrained structure by H-NS). But Rif would not be expected to lower the abundance of NAPs around the genome. The parsimonious explanation is that supercoil density was restored once transcription was reduced by Rif during the incubation at 30°. In our view, non-supercoil factors might account for 2–3 fold of a 50-fold impact that a *gyrB1820* mutation exerts on resolution. An independent measure of chromosomal supercoil structure at specific locations would be a very useful tool to help resolve the issue.

### Models for Regulating Supercoil Density

Various theories for regulating chromosomal supercoiling have been proposed since gyrase was discovered and the importance of negative supercoiling was revealed [59]. One model is that cells maintain a uniform level of supercoiling throughout the chromosome by varying levels of gyrase and Topo I [60]. When chromosomes experience a significant supercoil decline, the change is sensed by the promoter of GyrB, which increases expression by about 2-fold along with about 100 other ORFs [12]. When excessive levels of (−) supercoiling accumulate, transcription from the Topo I promoter [61] increases along with about 200 other ORFs [11], [62]. This system clearly modulates expression of GyrB and Topo I. However, because supercoil levels do not respond to changes in enzyme levels in a dramatic way, we view this as a fine tuning system [63]. For example, increasing or decreasing the abundance of Topo I or gyrase by 10% resulted in only a 1.3% change in DNA supercoil density [64], which would be equivalent to a MIF of 1.05 or 0.95 for each 10% difference in enzyme level. By contrast, when the *Salmonella* GyrB protein was expressed at 10% of normal levels in WT *E. coli*, the toxic effect caused the disappearance of most cells containing the plasmid [10]. We speculate that 50 slow or uncoordinated chimeric gyrase variants working in a WT background may cause sporadic supercoil disruptions with toxic consequences, perhaps by promoting RNAP blockades to the fast moving replisomes.

A second model proposes that a long range supercoil gradient exists within the bacterial chromosome [65]. The origin of replication was proposed to have the highest supercoiling level with σ = −0.068, and the terminus was predicted have the lowest σ = −0.043 with a smooth transition along the genome [65]. If constrained and diffusible supercoiling densities partition equally along the gradient, this model predicts resolution efficiencies of the supercoil sensor to decline from 75–80% near oriC to 15–20% at the terminus. However, our data disagree with this model. Our resolution assays showed equal recombination in 5 different macrodomains. Moreover, previous investigators used supercoil-responsive promoter fusions to *lacZ* and *luxAB* to test supercoiling levels in both the *E. coli* and *Salmonella* chromosomes [66]–[67]. They both found uniform levels of supercoiling along the genome, although none of their test positions were located close to highly transcribed genes or the *dif* site.

Our data suggest that the Liu and Wang model of twin domains of local opposite-handed supercoiling is a dominant force near the 30–50 highly transcribed genes [13]. Although the impact of transcription may be limited to a 10 kb zone from the point of origin, like transpositions immunity in Mu [68], a persistent loss of supercoil density during transcription can spread, causing slight or dramatic relaxation of chromosome DNA structure, depending on how an allele modifies gyrase supercoiling efficiency. We propose that the impact of RNA polymerase on global supercoil density is linked to transcription speed. At 30° in WT *Salmonella*, the elongation rate ranges from 45–60 nt/sec at different points around the genome (Figure 5). This causes DNA rotations of 4–6 supercoils per second. WT gyrase processively supercoils DNA at 4–5 sc/sec at 30° (Rovinskiy and Higgins, manuscript submitted) and Topo I removes negative supercoils at this rate in single molecule studies. Any condition that reduces gyrase supercoiling without directly reducing transcription kinetics or Topo I activity would cause supercoil density to decline across the genome.

We were surprised that a TS mutant of Topo IV also lost significant negative supercoiling at the permissive growth temperature. The common wisdom is that Topo IV functions primarily at the end of replication to decatenate sister chromosomes and allow complete segregation [7]. However, recent work shows that the C-terminal domain of Topo IV interacts with the hinge region of the MukB condensin [68], implicating Topo IV in processes occurring near the fork. Perhaps, in conjunction with DNA compaction, Topo IV removes (+) supercoils of transcription to prevent disruptive interactions between replisomes and RNA polymerase [69]–[70].

### Complex Regulation of the Transcription/Translation System

The third piece of experimental evidence supporting the mechanistic linkage between rates of gyrase and RNAP catalysis is the decreased rate of coupled transcription/translation throughout the chromosome in cells carrying a *gyrB1820* mutation (Figure 5.) The mean WT transcription rate was 52±11 nt/sec for the 7 sites, which fell to 32±6 nt/sec in GyrB1820 strains. We propose that there is a strong selection for matching catalytic rates of gyrase supercoiling with transcription elongation. When cells have a sluggish gyrase, transcription/translation slows down. In cells with reduced transcription efficiencies, like the *rpoC* Δ215–220, WT gyrase boosted supercoiling above the level in WT cells (Figure 4). Excess supercoiling wastes ATP, increases the likelihood that cells form toxic R-loops at locations of high transcription, and increases the susceptibility of chromosomal DNA to oxidative damage by free radicals that attack single stranded regions more efficiently than double stranded DNA.

Many components are now known to contribute to the transcription/translation enterprise. The list of factors includes DksA, NusA, NusG, MFD, Rho, RfhA, GreA, GreB, RNAP, ppGpp, tmRNA, Topo I, cAMP, cyclic GMP, and ribosomes. Interestingly, when the Cozzarelli lab set up a genetic screen to identify genes that might encode “domainins,” i.e. proteins controlling supercoil density, they uncovered a surprise gene, *dksA*, in addition to genes for the expected NAPs [28]. DksA mediates the stringent response by binding to RNA polymerase and placing ppGpp near the catalytic active site [71]. DksA makes sense in our model, because it changes transcription rates under stringent conditions. We recently tested deletions in GreA and GreB, which are proteins that promote processive transcription and salvage polymerases that have stalled in mid-stream. Mutants of both subunits raised the average supercoil density of *Salmonella* (Chesnokova and Higgins, unpublished results). These observations, along with older experiments showing that mutations in RNA polymerase influence cellular resistance to the gyrase inhibitors novobiocin and nalidixic acid [72], increase our confidence that RNA polymerase and its associated factors play a central role with gyrase in controlling the global supercoiling average.

### Does Supercoiling Control Transcription or Is It the Other Way Round?

DNA supercoiling has generally been studied as a mechanism to control gene expression by modulating promoter activity around the chromosome [11], [73]. *In vivo*, 300 *E. coli* genes are reported to change expression within 5 min after DNA relaxation by drug treatment [12]. However, three problems with studying supercoil regulation of transcription are often ignored or not considered important. First, transcription increases upstream and decreases downstream supercoil levels respectively, so the act of transcription would put the promoter in a zone of increased supercoil density. The increase in supercoiling is substantial [13], so after 200 *E. coli* genes are induced by increased (−) supercoiling, a different mechanism would be needed to turn them off.

Second, most of the chromosomal changes in transcription detected after DNA relaxation are 2-fold differences. The transcription rate of the Lac operon, which varies several hundred fold from the uninduced state to maximum expression, also changes elongation rate by 2-fold, according to its chromosome position (Figure 5). This is not a result of genetic adaptation, but is dictated by local differences in chromosome dynamics. Dissecting 2-fold changes in gene expression is a daunting task and can be the result of 3 different 1.3-fold causes. It is unclear how many of the 300 *E. coli* supercoil responsive genes actually improve fitness and how many represent regulatory noise that is insignificant from a physiological perspective.

Third, many investigators rely on plasmids to estimate chromosomal supercoiling and to gauge the effects of mutations on chromosomal structure. But plasmids can be misleading. By assuming that pUC19 was a good reporter of chromosome supercoil density, we completely missed the impact of transcription and the large supercoiling change in a *Salmonella gyrB652* mutant chromosome [26]. pUC19 lacks strong promoters and when plasmids from WT and *gyrB652* strains were compared, they differed by only 1 topoisomer. One cause for these chromosome/plasmid differences is that plasmids are single domain elements, except when they have an anchoring element or two active transcription units moving in opposing polarities [33]. In single domain plasmids, positive and negative supercoils cancel out by diffusing around the circle [74]. For plasmids with strong promoters, the primary topological effects are changes in constrained supercoil density associated with each added RNA polymerases [75]–[76].

### Implications and Further Experiments

*E. coli* and *Salmonella* have a 15% supercoil difference that changes the phenotype of multiple proteins contributing to chromosome dynamics [10]. Could species-specific amino acid substitutions in gyrase orthologs fine-tune supercoil densities in these closely related organisms? Recent work suggests this could be the case. One difference between the *E. coli* and *Salmonella* GyrA proteins is the amino acid sequence and length of the acidic amino acid-rich C-terminal tail (Figure S2). This C-terminal segment controls DNA looping of the pinwheel domain and establishes the supercoil reverse point for *E. coli* gyrase [77]. Moreover, when the *E. coli* GyrA ortholog was compared to *M. tuberculosis* (*M. tb.*) GyrA, the latter protein lacked C-terminal features present in *E. coli* [78]. *In vitro* supercoiling tests confirmed that both the speed and endpoint of M. *Tb* gyrase supercoiling are lower than those measured for the *E. coli* enzyme. Secondly, the GyrB subunit has the ATP binding site that fuels the supercoiling reaction. Whereas *Salmonella* GyrB protein is toxic in *E. coli*, the reverse is not true. *Salmonella* tolerates the *E. coli* GyrB chromosomal substitution, and the average supercoiling level of this strain increased at multiple chromosomal locations, including the region immediately downstream of *rrnG* (Rovinskii and Higgins, unpublished data.) Therefore, both gyrase subunits contribute to the enzyme v_max_ and supercoil endpoint.

Three untested issues related to these finding are worth mentioning. They involve current limitations on the fluxuation of supercoil density that we can monitor, the implications of the *Salmonella/E. coli* comparison for other bacterial species, and the relevance of this work to gene expression in eukaryotes. First, our data represent the mean values of an ensemble of cells in different states of the cell cycle during rapid division in rich medium and during slower growth in medium containing a defined carbon sources. Many investigators assume that supercoil density in a bacterium is maintained at a static modulus so that small changes in supercoil density can be used to modulate gene expression. Our view is more dynamic. The constant thrust from highly transcribed genes causes local gradients of supercoil density to arise throughout the genome. If topoisomerase efficiency is changed by metabolism or mutation, supercoil change spreads across the genome. However, little is currently known about single cell metabolism. For example, yeast cells go through an ultradian cycle that oscillates between periods of reductive reactions of the TCA cycle followed by an oxidative phosphorylation phase that increases ATP concentration [79]–[80]. Expression of most yeast genes increases then declines in either the oxidative period (a few genes) or the reductive phase (most genes). This cycle is shorter than the cell cycle and it is usually studied in carbon- or phosphate-limited chemostats, where yeast self-synchronize with the acetate flux. But non-synchronized cells show the same periodic variations of gene expression [81]. Bacteria could have similar behavior because they share with yeast a mechanism to increase or decrease acetate metabolism using a sirtuin-dependent acetylation/deacetylation of acyl-CoA synthase [82]. A test of cyclical transcription and negative supercoiling pulses for periods shorter than a bacterial cell cycle is challenging and would require different approaches.

The second interesting issue is the relationship between optimum growth rates and supercoil levels in different bacterial species. A significant supercoiling difference exists between *E. coli* and *Salmonella* [10]. WT *E. coli* cells grow faster than *Salmonella* and they double every 25 min at 37° in rich medium where transcription elongation rates top out at 90 nt/sec [83]. The fastest doubling time for *Caulobacter crescentus* is 2 h [84], presumably because rapid growth rates are not important for life in open ocean water. *M. tb*. has a doubling time of 14 h, contains only 1 ribosomal RNA operon, and encodes a gyrase with significantly slower v_max_ and a lower supercoiling endpoint than *E. coli* [78]. To understand chromosome structure in prokaryotes other than *E. coli* and *Salmonella*, methods will be needed to measure in vivo transcription/translation rates and to define supercoil density at multiple chromosome locations.

Third, might a pattern of covariant tempos of transcription elongation and topoisomerase turnover apply to eukaryotes? The short answer seems to be yes. In yeast, a type 1B topoisomerase (Topo I) relaxes both positive and negative supercoils and is active during transcription. Single molecule studies [85] showed that yeast Topo I relaxes both (+) and (−) supercoils at ≥4 sc/sec, matching the yeast Pol II transcription elongation speed of 30–40 nt/sec [86]. Camptothecin caused little change in the Topo I-dependent relaxation of (−) supercoils, but the rate of (+) supercoil removal fell 40-fold in the presence of drug. When the topology of *in vivo* transcribed DNA was analyzed, camptothecin treated yeast cells produced highly (+) supercoiled DNA, and further transcription was impeded [85], [87]. This is similar to the behavior we see in gyrase mutants (Figure 2). Therefore, tuning transcription machinery to topoisomerase catalytic rates may be necessary for efficient gene expression in yeast as well as in other eukaryotes.

## Materials and Methods

### Strain Construction

All strains in this work are derivatives of S. Typhimurium LT2, and their genotypes are listed in Table 1. Insertion mutagenesis was done using the λ Red recombineering method and the plasmids pSIM5 or pSIM6 [88]–[89]. The PCR amplification of drug modules for insertion into the chromosome and the electroporation conditions used to introduce DNA for homologous recombination were carried out as described previously [31].

Chromosomal recombinants were selected as antibiotic-resistant colonies on LB medium or as Lac^+^ colonies on minimal lactose medium. In each case the expected recombinant genotype was verified by PCR analysis using flanking PCR primers. Each recombinant was tested and shown to contain a cassette-modified allele with no WT allele present. Transduction crosses were performed as described previously using P22 HT105/1 int-201, a high-efficiency transducing variant of bacteriophage P22 [20].

The growth rate of individual strains was measured in early-mid log phase and calculated from the log slope of change over time of the OD_650_ between 0.01 and 0.4. Each strain was tested, starting from three independent colonies grown overnight and diluted 100 fold in fresh LB at 30°. Results are reported ±1 standard deviation from the average.

### Plasmids

Plasmid pJB γδ 30′ was used to induce the expression of a modified form of γδ resolvase. In this plasmid, resolvase is controlled from the λ*P*_L_ promoter using the TS cI857 repressor [21]. In pJB γδ 30′, 11 residues were incorporated at the natural C-terminus of resolvase that makes an SsrA degradation tag, which targets the protein to degradation by the ClpXP proteosome. At position 9 of the 11 amino acid SsrA tag, a L9D substitution gives the protein a 30 min half-life in exponentially growing *E. coli* and *S.* Typhimurium cells [21].

### Resolution Assays

Log-phase cultures growing in LB at 30° were sampled at a density of 50 Klett units. A 0.1 ml aliquot of each culture was placed in a 42°C shaking water bath for 10 min to induce Resolvase expression. The induced cells were immediately diluted with 2 ml of LB+Cm and incubated overnight at 30°C. On the following day, 100 µl aliquots of 10^−6^ dilutions of each culture were plated on LB medium or on NCE glucose minimal medium containing chloramphenicol and 5-bromo-4-chloro-3-indolyl β-D-galactoside (X-gal) plus 200 µM IPTG [13]. Plates were incubated for 2–3 days at 30°, and deletion frequencies were scored by counting the number of white colonies that reveal the loss of *lacZ* [20]. Each data point represents the average ±1 standard deviation of at least three independent experiments in which ≥200 colonies were counted for drug sensitivity or loss of lacZ expression.

### Transcription Elongation Assays

To measure elongation rates in *Salmonella*, the β-Gal method described by Vogel was used [55]. A flask with 20 ml of minimal AB medium supplemented with glucose [90] was inoculated with 2 ml of a fresh overnight culture, and growth was carried out at 30° or 37°. Samples (0.5 ml) of each culture having an OD_600_ between 0.2–0.4 were added to 500 ul ZS buffer (chilled at 4°C) containing 200 ug/ml chloramphenicol. Three samples were taken at 10 s intervals for the background measurement, and IPTG at a final concentration 1.5 mM was added to each culture. Aliquots of 500 ul were withdrawn every 10 sec and mixed with 500 µl ZS buffer for about 4 min. 100 µl chloroform was added to each sample followed by 200 ul ONPG which initiated a timed enzyme reaction. Reactions incubated at 30° for 1.5–4 hrs to allow development of appropriate levels of color were stopped by the addition of 500 ul Na_2_CO_3_. The OD_420_ and OD_550_ values were taken, and standard Miller Units were calculated as described [91]. Lag times and the transcription rates were determined using three independent colonies for each strain with results reported as the average value ±1 SD of the mean.

### Deletion of the *rpoC* Region 215–220

To make a 6 amino acid deletion (ΔKKLTKR) in the *rpoC* gene of *Salmonella*, we used the method described by Sharan et al. [92]. Four primers were designed with a 20 bp overlap of N- and C- terminal segments of RpoC. The primer pair of Rpoc fwd (CGCGAAGATGGGGGCGGAAG) and RpoC rev del (aaggcttccagcagtttgat acgcttggtttcggagttgg) and RpoC frd del2 (ccaactccgaaaccaagcgt atcaaactgctggaagcctt) and Rpoc rev (CCATCCAGCGGAACCAGCGG) both make 130 bp PCR products carrying the upstream and downstream region of RpoC with a deletion in both fragments. These products were combined and amplified with the RpoC fwd and RpoC rev primers to generate a 220 bp PCR with the 6 amino acid deletion at the center. The PCR DNA was introduced by electroporation of WT LT2, which had been pre-induced for recombineering function encoded on the pSIM6 plasmid. After incubation for 2 hrs in LB to allow recovery, 200–400 cells were plated onto 5 LB plates and incubated at 30° for 2 days. Small colonies were observed in both WT and fis mutant plates at a frequency of 1/500 to 1/1000. Three colonies were picked and subjected to PCR sequencing using the outside primers and DNA template from a negative control. Every small colony we tested carried the deletion called RpoC (ß′ Δ115–220) by Bartlett et al. [54] and gave no WT RpoC sequence.

## Supporting Information

Figure S1 Strain construction for supercoil analysis in the *Salmonella* chromosome. In each strain, a single 34 bp Frt site was introduced into the chromosome using the λ red recombination methods [89]. In the diagram shown above, a Frt site was placed between the *atpI* gene and the *gidB* gene. The circular form of the Lac-Gn Res module isolated from a donor strain with a chromosomal copy by thermo-induction of the Flp recombinase was re-inserted in new locations by transformation of cells induced for the Flp expression (center). Each module was transferred by P22 transduction to strains with the desired test gene plus the pJB-γδ-Res-Ssra-L9D plasmid, which encodes a 30 min half-life resolvase.
(TIF)

Figure S2 Map of GyrA and GyrB subunits of *S. typhimurium* gyrase. In A the GyrA protein is shown with the catalytic tyrosine-122 (Y122*). The *gyrA213* ^TS^ mutation is caused by a change of Arg 358 to His in the DNA binding/cleavage domain (aqua), and the *gyrA209* ^TS^ allele changes Gly 597 to Asp in the second ß-propeller domain (Blue). There are 72 codon differences between WT *E. coli* and *Salmonella* GyrA (black hatches); most changes are in the carboxyl-terminal segment of the protein that involve DNA looping (blue) and regulation of looping by the acidic tail (Red) [77]. B. The two TS mutations of *gyrB* used in this study are the *gyrB1820* ^TS^ mutation of Cys 56 to Tyr in the ATPase domain (red) and the *gyrB652* ^TS^ substitution of Arg 436 to Ser in the magnesium binding/DNA cleavage activation domain (green). Only 28 amino acids have diverged between *E. coli* and *Salmonella* GyrB (black hatches.)
(TIF)

Figure S3 Western Blot analyses of Resolvase expression in WT and mutant strains of *Salmonella enterica*. Bacteria were grown to an optical density of 50 Klett units. Aliquots (4 ml) of each culture were harvested before and after temperature induction. Cells concentrated by centrifugation at 4°C were suspended in sterile 100 µl TGED buffer (50 mM Tris HCl pH 8.0, 10% glycerol, 1 mM EDTA pH 8.0 and 1 mM DTT). 8 µl aliquots were mixed with 2 µl 5× SDS PAGE loading buffer (250 mM Tris HCl pH 6.8, 500 mM DTT, 10% SDS, 0.5% bromophenol blue, 50% glycerol), boiled 5 minutes and spun down. 5 µl of each supernatant was loaded onto an SDS 15% polyacrylamide gel. Membranes washed twice in TBST and once in TBS (100 mM Tris HCl pH 7.5, 2.5 M NaCl) were developed using PerkinElmer Western Lightning *Plus*-ECL kit according to manufacturer recommendations. Two cell proteins run near the resolvase protein react with the rabbit antiserum; one lies above and one much lighter band runs at the same position as Resolvase (21 kDa) in the control lane. Lane 1) NH2002 (WT LT2) without a plasmid after 10 min at 42°. In all other lanes each strain has the pJBRES *30′*; 2) NH6000 (LT2 WT) uninduced; 3) NH6000 10 min at 42°; 4) NH6000 10 min at 42° followed by 30 min incubation in Rif at 30°; 5) NH6018 (*gyrA213*) uninduced; 6) NH6018 10 min at 42°; 7) NH6019 (*gyrA209*) uninduced; 8) NH6019 after 10 min at 42°; 9) NH6037 (*gyrB1820*) uninduced; 10) NH6037 10 min at 42°; 11) NH6206 (*rpoC*) uninduced; 12) NH6206 10 min at 42°.
(TIF)

Table S1 List and genetic structure of all strains used in this study. All strains were created for this or previous studies related to this work.
(DOC)
