# Supplementary figures and images for: Rates of Gyrase Supercoiling and Transcription Elongation Control Supercoil Density in a Bacterial Chromosome
Source: PLoS Genet. 2012 Aug 16;8(8):e1002845. doi: 10.1371/journal.pgen.1002845 (PMC3420936; doi:10.1371/journal.pgen.1002845)

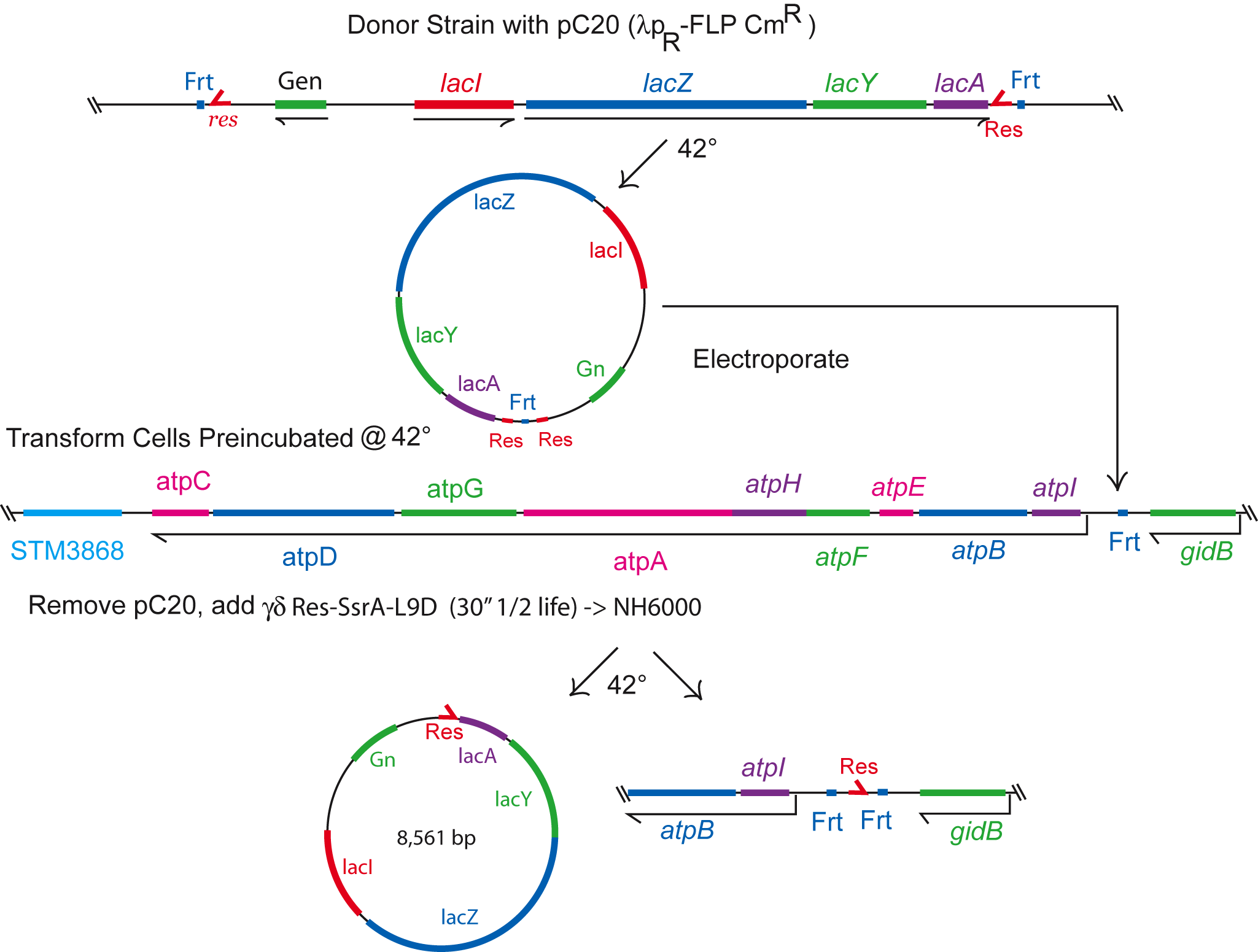

Supplement: Figure S1 — Strain construction for supercoil analysis in the Salmonella chromosome. In each strain, a single 34 bp Frt site was introduced into the chromosome using the λ red recombination methods [89]. In the diagram shown above, a Frt site was placed between the atpI gene and the gidB gene. The circular form of the Lac-Gn Res module isolated from a donor strain with a chromosomal copy by thermo-induction of the Flp recombinase was re-inserted in new locations by transformation of cells induced for the Flp expression (center). Each module was transferred by P22 transduction to strains with the desired test gene plus the pJB-γδ-Res-Ssra-L9D plasmid, which encodes a 30 min half-life resolvase. (TIF) [file pgen.1002845.s001.tif]

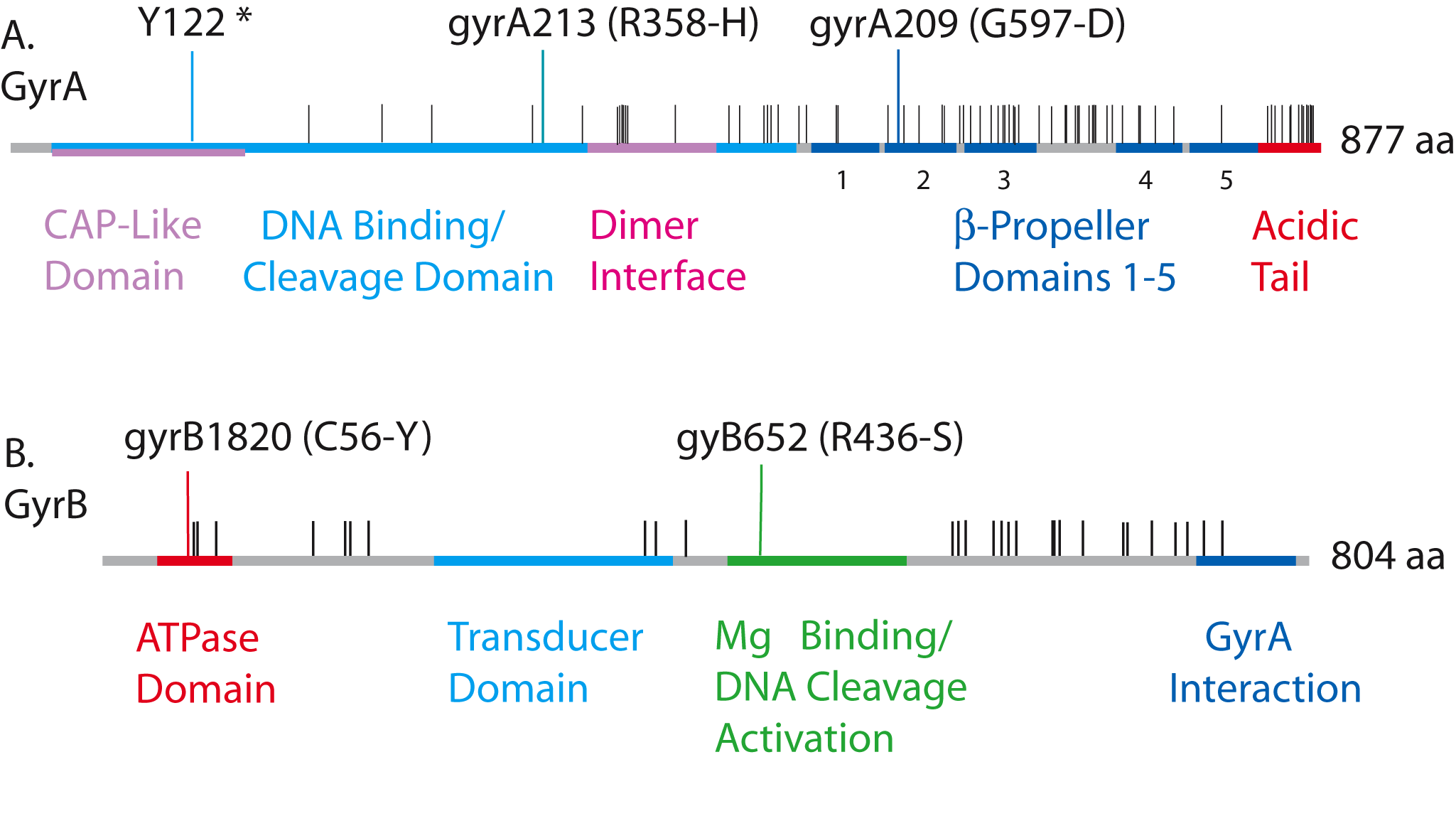

Supplement: Figure S2 — Map of GyrA and GyrB subunits of S. typhimurium gyrase. In A the GyrA protein is shown with the catalytic tyrosine-122 (Y122*). The gyrA213 TS mutation is caused by a change of Arg 358 to His in the DNA binding/cleavage domain (aqua), and the gyrA209 TS allele changes Gly 597 to Asp in the second ß-propeller domain (Blue). There are 72 codon differences between WT E. coli and Salmonella GyrA (black hatches); most changes are in the carboxyl-terminal segment of the protein that involve DNA looping (blue) and regulation of looping by the acidic tail (Red) [77]. B. The two TS mutations of gyrB used in this study are the gyrB1820 TS mutation of Cys 56 to Tyr in the ATPase domain (red) and the gyrB652 TS substitution of Arg 436 to Ser in the magnesium binding/DNA cleavage activation domain (green). Only 28 amino acids have diverged between E. coli and Salmonella GyrB (black hatches.) (TIF) [file pgen.1002845.s002.tif]

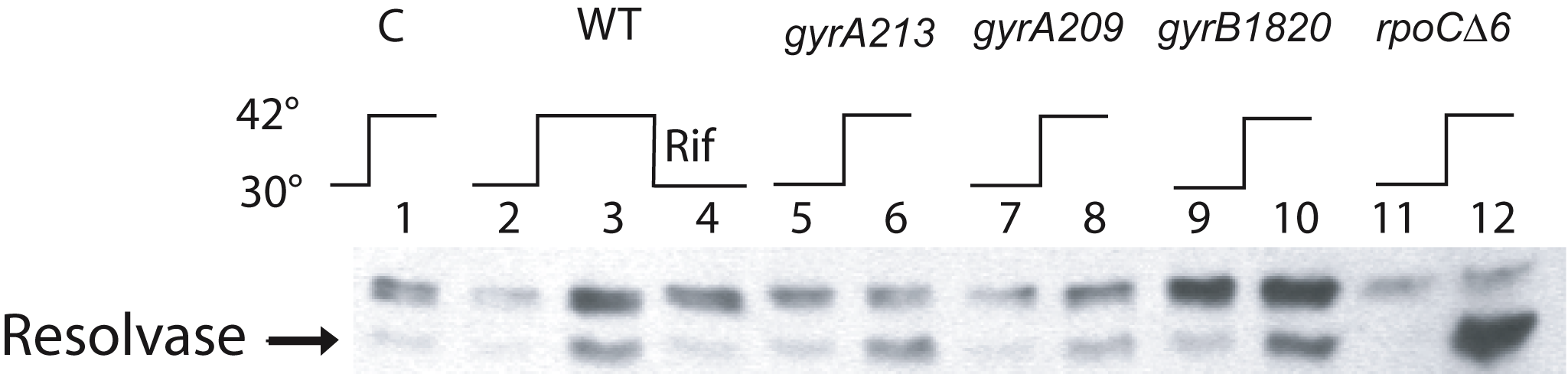

Supplement: Figure S3 — Western Blot analyses of Resolvase expression in WT and mutant strains of Salmonella enterica. Bacteria were grown to an optical density of 50 Klett units. Aliquots (4 ml) of each culture were harvested before and after temperature induction. Cells concentrated by centrifugation at 4°C were suspended in sterile 100 µl TGED buffer (50 mM Tris HCl pH 8.0, 10% glycerol, 1 mM EDTA pH 8.0 and 1 mM DTT). 8 µl aliquots were mixed with 2 µl 5× SDS PAGE loading buffer (250 mM Tris HCl pH 6.8, 500 mM DTT, 10% SDS, 0.5% bromophenol blue, 50% glycerol), boiled 5 minutes and spun down. 5 µl of each supernatant was loaded onto an SDS 15% polyacrylamide gel. Membranes washed twice in TBST and once in TBS (100 mM Tris HCl pH 7.5, 2.5 M NaCl) were developed using PerkinElmer Western Lightning Plus-ECL kit according to manufacturer recommendations. Two cell proteins run near the resolvase protein react with the rabbit antiserum; one lies above and one much lighter band runs at the same position as Resolvase (21 kDa) in the control lane. Lane 1) NH2002 (WT LT2) without a plasmid after 10 min at 42°. In all other lanes each strain has the pJBRES 30′; 2) NH6000 (LT2 WT) uninduced; 3) NH6000 10 min at 42°; 4) NH6000 10 min at 42° followed by 30 min incubation in Rif at 30°; 5) NH6018 (gyrA213) uninduced; 6) NH6018 10 min at 42°; 7) NH6019 (gyrA209) uninduced; 8) NH6019 after 10 min at 42°; 9) NH6037 (gyrB1820) uninduced; 10) NH6037 10 min at 42°; 11) NH6206 (rpoC) uninduced; 12) NH6206 10 min at 42°. (TIF) [file pgen.1002845.s003.tif]
