# Supplementary material for: Rates of Gyrase Supercoiling and Transcription Elongation Control Supercoil Density in a Bacterial Chromosome
Source: PLoS Genet. 2012 Aug 16;8(8):e1002845. doi: 10.1371/journal.pgen.1002845 (PMC3420936; doi:10.1371/journal.pgen.1002845)
Supplement: Table S1 — List and genetic structure of all strains used in this study. All strains were created for this or previous studies related to this work. (DOC) [file pgen.1002845.s004.doc]

## Table S1.

Strain Genotype Plasmid Cs

NH2002 LT2 WT

NH6000 *atpI* < Frt Res lac Gn Res Frt > *gidB* pJBRES *30’* 85

NH6001 *STM3261* < Frt Res *lac* Gn Res Frt > *STM3262* pJBRES *30’* 71

NH6002 *smpB* < Frt Res *lac* Gn Res Frt > *STM2689* pJBRES *30’*  58

NH6003 *STM2135* < Frt Res *lac* Gn Res Frt > *yegQ* pJBRES *30’* 45

NH6005 *STM1554* < Frt Res *lac* Gn Res Frt > *STM1553* pJBRES *30’* 33

NH6006 *STM0951* < Frt Res *lac* Gn Res Frt > *STM0952* pJBRES *30’* 21

NH6007 *ampH* < Frt Res *lac* Gn Res Frt > *sbmA* pJBRES *30’*  9

NH6008 *STM4442*::< Frt Res *lac* Gn Res Frt > pJBRES *30’* 96

NH6009 *marC*::< Frt Res *lac* Gn Res Frt > pJBRES *30’* 33

NH6010 *STM1612*::< Frt Res *lac* Gn Res Frt > pJBRES *30’* 35

NH6011 *ycjG*:: < Frt Res *lac* Gn Res Frt > pJBRES *30’* 36

NH6012 *acnA* < Frt Res *lac* Gn Res Frt > *cysB* pJBRES *30’* 37

NH6016 NH6000 *zeh754*::Tn*10 gyrA213* pJBRES *30’* 85

NH6018 NH6005 *zeh754*::Tn*10* *gyrA213*  pJBRES *30’* 85

NH6019 NH6000 *zeh754*::Tn*10* *gyrA209* pJBRES *30’* 85

NH6020 NH6001 *zeh754*::Tn*10* *gyrA209* pJBRES *30’* 71

NH6021 NH6002 *zeh754*::Tn*10* *gyrA209* pJBRES *30’*  58

NH6022 NH6003 *zeh754*::Tn*10* *gyrA209* pJBRES *30’* 45

NH6024 NH6005 *zeh754*::Tn*10* *gyrA209*  pJBRES *30’* 33

NH6025 NH6006 *zeh754*::Tn*10* *gyrA209* pJBRES *30’* 21

NH6026 NH6007 *zeh754*::Tn*10* *gyrA209* pJBRES *30’*  9

NH6027 NH6008 *zeh754*::Tn*10* *gyrA209* pJBRES *30’* 96

NH6028 NH6000 *zib6794*::Tn*10* *gyrB652* pJBRES *30’* 85

NH6029 NH6001 *zib6794*::Tn*10* *gyrB652* pJBRES *30’* 71

NH6030 NH6002 *zib6794*::Tn*10* *gyrB652* pJBRES *30’*  58

NH6031 NH6003 *zib6794*::Tn*10* *gyrB652* pJBRES *30’* 45

NH6033 NH6005 *zib6794*::Tn*10* *gyrB652*  pJBRES *30’* 33

NH6034 NH6006 *zib6794*::Tn*10* *gyrB652* pJBRES *30’* 21

NH6035 NH6007 *zib6794*::Tn*10* *gyrB652* pJBRES *30’*  9

NH6036 NH6008 *zib6794*::Tn*10* *gyrB652* pJBRES *30’* 96

NH6037 NH6000 *zib748*::Tn*10* *gyrB1820* pJBRES *30’* 85

NH6040 MH6000 *zgc2393*::Tn*10 parC281* pJBRES *30’* 85

NH6043 NH6000 *zgc2393*::Tn*10* *parE206* pJBRES *30’* 85

NH6044 NH6001 *zgc2393*::Tn*10 parE206* pJBRES *30’* 71

NH6045 NH6002 *zgc2393*::Tn*10* *parE206* pJBRES *30’*  58

NH6046 NH6003 *zgc2393*::Tn*10* *parE206*  pJBRES *30’* 45

NH6048 NH6005 *zgc2393*::Tn*10* *parE206*  pJBRES *30’* 33

NH6049 NH6006 *zgc2393*::Tn*10* *parE206*  pJBRES *30’* 21

NH6056 NH6007 *zgc2393*::Tn*10 parE206*  pJBRES *30’*  9

NH6058 NH6008 *zgc2393*::Tn*10* *parE206*  pJBRES *30’* 96

NH6072 STM2655:: < Frt Res *lac* Gn Res Frt > pJBRES *30’* 57.64

NH6073 *clpB* < Frt Res *lac* Gn Res Frt > *rrlG* pJBRES *30’* 57.65

NH6108 NH6001 *zib748*::Tn*10* *gyrB1820* pJBRES *30’* 71

NH6109 NH6002 *zib748*::Tn*10* *gyrB1820* pJBRES *30’*  58

NH6110 NH6003 *zib748*::Tn*10* *gyrB1820* pJBRES *30’* 45

NH6111 NH6005 *zib748*::Tn*10* *gyrB1820*  pJBRES *30’* 33

NH6112 NH6006 *zib748*::Tn*10* *gyrB1820* pJBRES *30’* 21

NH6113 NH6007 *zib748*::Tn*10* *gyrB1820* pJBRES *30’*  9

NH6114 NH6008 *zib748*::Tn*10* *gyrB1820* pJBRES *30’* 96

NH6118 NH6009 *zib748*::Tn*10* *gyrB1820* pJBRES *30’* 33’

NH6119 NH6010 *zib748*::Tn*10* *gyrB1820* pJBRES *30’* 35

NH6120 NH6011 *zib748*::Tn*10* *gyrB1820* pJBRES *30’* 36

NH6121 NH6012 *zib748*::Tn*10* *gyrB1820* pJBRES *30’* 37

NH6222 NH6000 *rpoC* (β' Δ215-220) pJBRES *30’* 85

NH6223 NH6001 *rpoC* (β' Δ215-220) pJBRES *30’* 71

NH6224 NH6002 *rpoC* (β' Δ215-220) pJBRES *30’*  58

NH6225 NH6003 *rpoC* (β' Δ215-220) pJBRES *30’* 45

NH6226 NH6005 *rpoC* (β' Δ215-220) pJBRES *30’* 33

NH6227 NH6006 *rpoC* (β' Δ215-220) pJBRES *30’* 21

NH6228 NH6007 *rpoC* (β' Δ215-220) pJBRES *30’*  9

NH6229 NH6008 *rpoC* (β' Δ215-220) pJBRES *30’* 96

NH6230 NH6073 *rpoC* (β' Δ215-220) pJBRES *30’* 57.65

NH6231 NH6072 *rpoC* (β' Δ215-220) pJBRES *30’* 57.64
